# Geometric evaluation and quantifying dosimetric impact of diverse deformable image registration algorithms on abdomen images with biomechanically modeled deformations

**DOI:** 10.1002/acm2.14511

**Published:** 2024-09-11

**Authors:** Yilin Liu, Pengpeng Zhang, Jun Hong, Sadegh Alam, LiCheng Kuo, Yu‐chi Hu, Wei Lu, Laura Cerviño

**Affiliations:** ^1^ Department of Medical Physics Memorial Sloan Kettering Cancer Center New York New York USA

**Keywords:** abdomen imaging, biomechanically modeled deformations, DIR, dosimetry, patient‐specific QA

## Abstract

**Purpose:**

Deformable image registration (DIR) has been increasingly used in radiation therapy (RT). The accuracy of DIR algorithms and how it impacts on the RT plan dosimetrically were examined in our study for abdominal sites using biomechanically modeled deformations.

**Methods:**

Five pancreatic cancer patients were enrolled in this study. Following the guidelines of AAPM TG‐132, a patient‐specific quality assurance (QA) workflow was developed to evaluate DIR for the abdomen using the TG‐132 recommended virtual simulation software ImSimQA (Shrewsbury, UK). First, the planning CT was deformed to simulate respiratory motion using the embedded biomechanical model in ImSimQA. Additionally, 5 mm translational motion was added to the stomach, duodenum, and small bowel. The original planning CT and the deformed CT were then imported into Eclipse and MIM to perform DIR. The output displacement vector fields (DVFs) were compared with the ground truth from ImSimQA. Furthermore, the original treatment plan was recalculated on the ground‐truth deformed CT and the deformed CT (with Eclipse and MIM DVF). The dose errors were calculated on a voxel‐to‐voxel basis.

**Results:**

Data analysis comparing DVF from Eclipse versus MIM show the average mean DVF magnitude errors of 2.8 ± 1.0  versus 1.1 ± 0.7 mm for stomach and duodenum, 5.2 ± 4.0  versus 2.5 ± 1.0 mm for small bowel, and 4.8 ± 4.1  versus 2.7 ± 1.1 mm for the gross tumor volume (GTV), respectively, across all patients. The mean dose error on stomach+duodenum and small bowel were 2.3 ± 0.6% for Eclipse, and 1.0 ± 0.3% for MIM. As the DIR magnitude error increases, the dose error range increase, for both Eclipse and MIM.

**Conclusion:**

In our study, an initial assessment was conducted to evaluate the accuracy of DIR and its dosimetric impact on radiotherapy. A patient‐specific DIR QA workflow was developed for pancreatic cancer patients. This workflow exhibits promising potential for future implementation as a clinical workflow.

## 1 INTRODUCTION

Deformable image registration (DIR) has emerged as a valuable tool in radiation therapy (RT) for the evaluation of anatomical deformations.^1^ In the context of treatment planning, DIR plays a crucial role in assessing previous treatments through dose accumulation.^2^ For instance, it enables the registration of planning computed tomography (CT) scans with re‐simulation CT scans or images from different modalities such as weekly cone‐beam CTs or magnetic resonance imaging (MRIs) used in IGRT.^3^ This registration allows for the estimation of the cumulative dose resulting from multiple irradiation sessions. During the course of treatment, DIR finds application in image‐guided RT for contour propagation and verification of dose distribution using daily on‐treatment imaging.^4^ Moreover, DIR proves valuable in adaptive planning by assisting the determination of the accumulated dose from fraction to fraction in both external beam RT and brachytherapy, based on the evolving anatomical changes of the patient. Finally, DIR plays a role in post‐treatment assessment, including dose reporting of brachytherapy and toxicity prediction in voxel‐wise population analysis. By accurately mapping the delivered dose and assessing its impact on specific regions, DIR aids in understanding the treatment outcome and potential side effects at a more granular level.

With the increasing utilization of DIR in RT, it becomes crucial to reliably evaluate the performance of DIR algorithms to ensure their effective application. Inaccurate DIR results can lead to deviations between the delivered dose and the optimized planning dose, potentially resulting in overdosing of organs at risk (OAR). Various evaluation methods have been proposed in the literature to address this issue.

One commonly used approach for evaluating DIR accuracy is the volume‐based criterion, which involves comparing DIR‐generated contours with radiation oncologists (MD) drawn contours.^5^ Skornitze et al. assessed the performance of both rigid registration and DIR algorithms through qualitative ratings provided by radiologists.^6^ However, such comparisons do not provide precise information regarding the registration's local accuracy and precision in terms of anatomical “point‐to‐point” correspondence. To quantify local accuracy and precision of DIR while considering the intra‐observer error, landmarks manually placed by two observers can be employed.^7^ Chandler et al. conducted validation studies of rigid and deformable algorithms for motion correction in the lung and liver using defined landmarks. However, their evaluation was primarily visual, and only a limited number of landmarks (center of tumor and organs) were defined.^8^, ^9^ In another study, Huang et al. evaluated a DIR algorithm using Siemen Healthineers' Syngo commercial platform. The evaluation was performed according to the recommendations of the American Association of Physicists in Medicine (AAPM) Task Group Report 132 (TG‐132).^10^ To assess the algorithm, two physical phantoms were utilized: a deformable abdomen phantom that simulated respiratory‐induced deformation and a rigid phantom that simulated sliding motion between the chest wall and lungs during respiration.^11^ However, applying TG‐132 recommendations to the Syngo deformable registration algorithm posed challenges because the software lacked validation tools beyond image blending and did not provide the capability to export the deformed image set or the deformation vector field (DVF).

Although many efforts have been made, it is still challenging to evaluate DIR performance. Additionally, the geometric uncertainties associated with DIR significantly influence the accuracy of deformable dose accumulation. Cunliffe et al. proposed establishing a non‐linear relationship between DIR and accumulated dose uncertainties,^12^, ^13^ but the selection of the dosimetric parameters to be input for the non‐linear model is a major challenge in this study. Bohoudi et al. developed a model for evaluating DIR in dose accumulation, which involved an empirical selection of a combination of mathematical functions or an estimation of the model through machine learning techniques.^14^ Nevertheless, this model requires careful validation and analysis to ensure its accuracy and reliability. Alternatively, Niu et al. employed deformable phantoms composed of radiosensitive gels to assess the impact of DIR on dose mapping.^15^ However, the use of gels lacks the anatomical details necessary for a comprehensive evaluation of DIR on real anatomical structures and accurate dose accumulation. Moreover, McDonald et al. proposed the application of DIR in dose accumulation for MR‐guided adaptive radiotherapy and evaluated the impact of DIR on dose accumulation. However, in this study, only the comparison between adaptive doses and planned doses on pre‐treatment anatomy was performed to determine the doses received by OAR. The estimation of dose mapping errors was not conducted due to the absence of a ground truth reference.^16^

Several factors influence the quality and stability of DIR for clinical applications. These factors include inter‐user and intra‐user variability resulting from differences in user experience, the choice of registration algorithms, and the quality of input images.^10^ However, the lack of a reliable ground truth poses the most significant challenge in evaluating DIR, particularly in the abdomen. Images in this region often exhibit poor soft tissue contrast, and significant motion artifacts caused by physiological changes.

In our study, we introduced a novel patient‐specific quality assurance (QA) process aimed at evaluating the accuracy of DIR and its impact on dosimetry. To facilitate this evaluation, we employed a deformable digital phantom, ImSimQA (Oncology System Limited, Shrewsbury, UK), which is recommended by AAPM TG‐132 for DIR testing. The deformation of the digital phantom was generated with a realistic biomechanical model constructed based on patient's CT images. The evaluation process was conducted on two widely used commercial platforms: Eclipse v15.5 (Varian Medical Systems, Palo Alto, California, USA) and MIM v6.9.7 (MIM Software Inc., Cleveland, Ohio, USA).

## 2 MATERIALS AND METHODS

### 2.1 Patient‐specific QA workflow

In accordance with the guidelines of AAPM TG‐132, we propose a novel patient‐specific QA process aimed at assessing the accuracy of DIR on a per‐patient basis. One of the main challenges in DIR evaluation lies in obtaining reliable ground truth for the registered deformation. To tackle this challenge, we categorized the ground truth motion into respiratory motion and digestive motion to obtain them separately.

**FIGURE 1:**
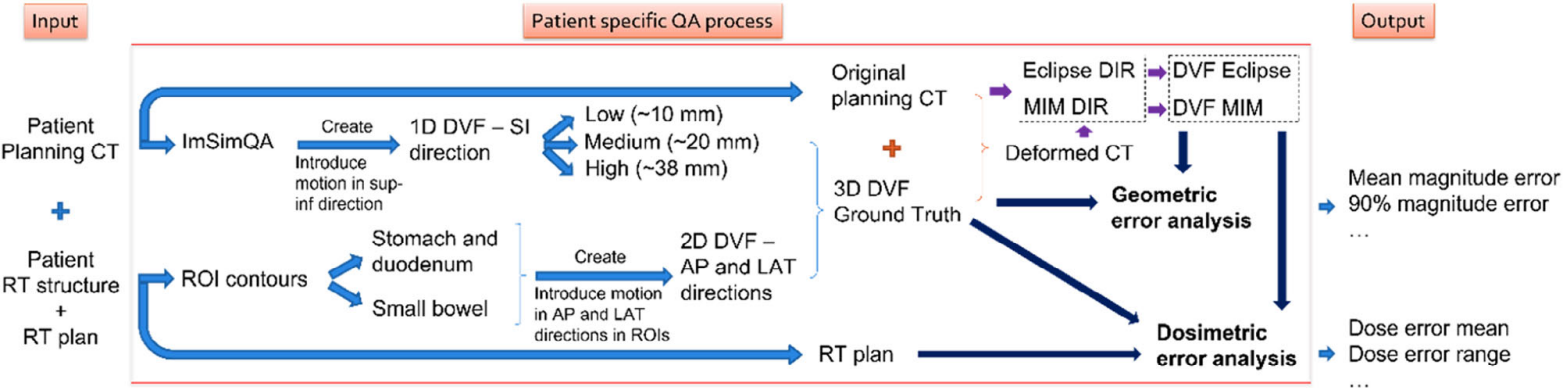
Workflow of the patient‐specific DIR QA process.

The workflow of the patient‐specific QA process for DIR evaluation is illustrated in Figure 1. First, we employed a realistic biomechanical model embedded in the TG‐132 recommended virtual phantom, ImSimQA (Shrewsbury, UK), to simulate respiratory motion. For each patient under evaluation, we generated a ground truth respiratory motion by deforming the planning CT image from the end of exhalation to the end of inhalation using ImSimQA. The biomechanical model initiated motion in the superior‐inferior (SI) direction in the lung and propagated it to the abdominal region, including the gross tumor volume (GTV) in the pancreas. ImSimQA offered simulated motions with low (10 mm), medium (20 mm), and high (38 mm) magnitudes, with the high magnitude being selected for our study.

Second, we introduced 5 mm translational motion in both the anterior‐posterior (AP) and lateral (LAT) directions to selected regions of interest (ROI), such as the stomach+duodenum and small bowel, to simulate digestive motion. The relevant RT structures were exported and converted into binary masks. The digestive motion was uniformly applied to all voxels within these masks. As a pilot study, we employed a simplified digestive motion model to represent random involuntary digestive motion. The magnitude of the deformations added to the ROI was determined based on the motion magnitudes observed in daily abdominal cone‐beam CT (CBCT) images. By combining the motion vectors in the AP and LAT directions with the SI DVF obtained from ImSimQA, we generated and introduced a 3D ground truth DVF to the CT images of the patient under evaluation. This 3D DVF represented realistic respiratory motion and simplified digestive motion.

The generated 3D DVF was then applied to the planning CT to generate a ground truth deformed CT. For our study, we utilized this synthetic ground truth 3D DVF, the planning CT, the corresponding ground truth deformed CT, and one set of RT structures which were contoured by certified physicians (MD) as the inputs to evaluate the performance of the DIR algorithms implemented in commercial platforms such as Eclipse v15.5 (Varian Medical Systems, Palo Alto, California, USA) and MIM v6.9.7 (MIM Software Inc., Cleveland, Ohio, USA). The DIR in Eclipse is the Smart Adapt (v15.5), which is integrated into the Image Registration module of Eclipse. MIM DIR we used applies an intensity‐based DIR algorithm.

DIR was performed by first importing the original CT and deformed CT to Eclipse and MIM. Prior to DIR, a rigid registration was applied to align the bony structures, with a specific focus on the spine. This initial rigid registration served as a pre‐processing step to improve alignment before performing DIR. Since both the planning CT and deformed CT shared the same imaging modality and contrast, intensity‐based DIR algorithms were selected in Eclipse and MIM. In our study, this workflow was repeated for all subjects, which included five patients (pt1‐pt5) diagnosed with pancreatic cancer. The radiation therapy prescription is listed in Table 1.

**TABLE 1:** Radiation therapy prescriptions for all patients in our study.

Patient no.	Prescription	Prescription dose level 1 (cGy)	Dose painting	Prescription dose level 2 (cGy)	Prescription dose level 3 (cGy)
1	180cGy*25fx	4500	300cGy*25fx	7500	
2	250cGy*15fx	3750	450cGy*15fx	6750	
3	180cGy*25fx	4500	300cGy*25fx	7500	
4	250cGy*15fx	3750	450cGy*15fx	6750	
5	180cGy*25fx	4500	300cGy*25fx; 400cGy*25fx	7500	10 000

Dose painting (DP) listed in the fourth column is a radiation therapy (RT) strategy implemented with IMRT for patients with heterogeneous tumors delivering higher dose to radiation resistant regions and less to sensitive ones, thus aiming to maximize tumor control with limited side effects. Also known as simultaneous integrated boost (SIB). For our patients, dose painting delivers a lower (level 1 prescription of 45 Gy) dose to a larger PTV, while a higher (level 2 prescription of 75  or 67.5 Gy) dose to a smaller PTV, which is inside the larger PTV and further from OARs.

### 2.2 Patient‐specific DIR performance analysis

#### 2.2.1 Geometric error analysis

After the DIR was performed using Eclipse and MIM software, the resulting DVF was exported from Eclipse and MIM for comparison with the 3D ground truth DVF. The DIR magnitude error was calculated for each voxel by subtracting Eclipse or MIM DVF from the ground‐truth DVF. Magnitude error is defined by formula.^1^ All DVF sets were linearly interpolated to 1 mm*1 mm*1 mm resolution, and the dicom origin of all DVF sets were aligned. In order to evaluate the comparison between the DIR results from Eclipse and MIM with our 3D ground truth DIR, various metrics such as mean error and 90th percentile error were computed for the GTV, stomach‐duodenum, small bowel, and the entire CT volume for each patient.

$$\begin{array}{ccc} & & \mathrm{formula}(1):\mathrm{Magnitude}\;\mathrm{error}\\ & & =\sqrt{\begin{array}{c} {\left(\mathrm{Eclipse}\;\mathrm{or}\;\mathrm{MIM}\;\mathrm{DVFx}-\mathrm{ground}-\mathrm{truth}\mathrm{DVFx}\right)}^{2}\\ +{\left(\mathrm{Eclipse}\;\mathrm{or}\;\mathrm{MIM}\;\mathrm{DVFy}-\mathrm{ground}-\mathrm{truth}\mathrm{DVFy}\right)}^{2}\\ +{\left(\mathrm{Eclipse}\;\mathrm{or}\;\mathrm{MIM}\;\mathrm{DVFz}-\mathrm{ground}-\mathrm{truth}\mathrm{DVFz}\right)}^{2} \end{array}} \end{array}$$

define: DVFx = DVF in anterior posterior direction
DVFy = DVF in left right direction
DVFz = DVF in superior inferior direction

The absolute magnitude errors of the DIR, referred to as DIR magnitude errors, were mapped across the entire field of view (FOV) of the CT scan, including the OARs such as the stomach, duodenum, and small bowel. The distribution of vector magnitudes and DIR magnitude errors were analyzed using histograms. A quantitative analysis of the DVFs obtained from Eclipse, MIM, and the ground truth was performed, and the results were tabulated. The measurements included the maximum and mean vector magnitudes of the ground truth DVF, the mean DIR magnitude errors of Eclipse or MIM for the entire CT scan and ROIs, and the 90th percentile measurements of the distribution of DIR magnitude errors. Inter‐observer differences were assessed by comparing the results obtained by different users of Eclipse and MIM. A comprehensive report was generated as a QA record, demonstrating the performance of the DIR for the patients of interest.

#### 2.2.2 Dosimetric error analysis

We also assessed the dosimetric uncertainties introduced by the DIR in our clinical workflow using our novel patient‐specific DIR QA procedure. The inputs for this workflow included the original CT scan, ground‐truth deformed CT scan, and the DVF sets generated by Eclipse and MIM, as described in the previous section.

During the evaluation, new deformed CT scan sets were generated from deforming the original CT scan using the DVF sets generated by Eclipse and MIM. These deformed CT scan sets were labeled as dCT‐Eclipse and dCT‐MIM, respectively. The original treatment plan was recalculated on these newly generated deformed CT scans, with the same beam settings. The resulting dose maps were labeled as DM_Eclipse and DM_MIM, respectively. Additionally, the original treatment plan was also recalculated on the ground‐truth deformed CT scan as a reference, leading to a dose map labeled as DM_Ground‐truth.

**FIGURE 2:**
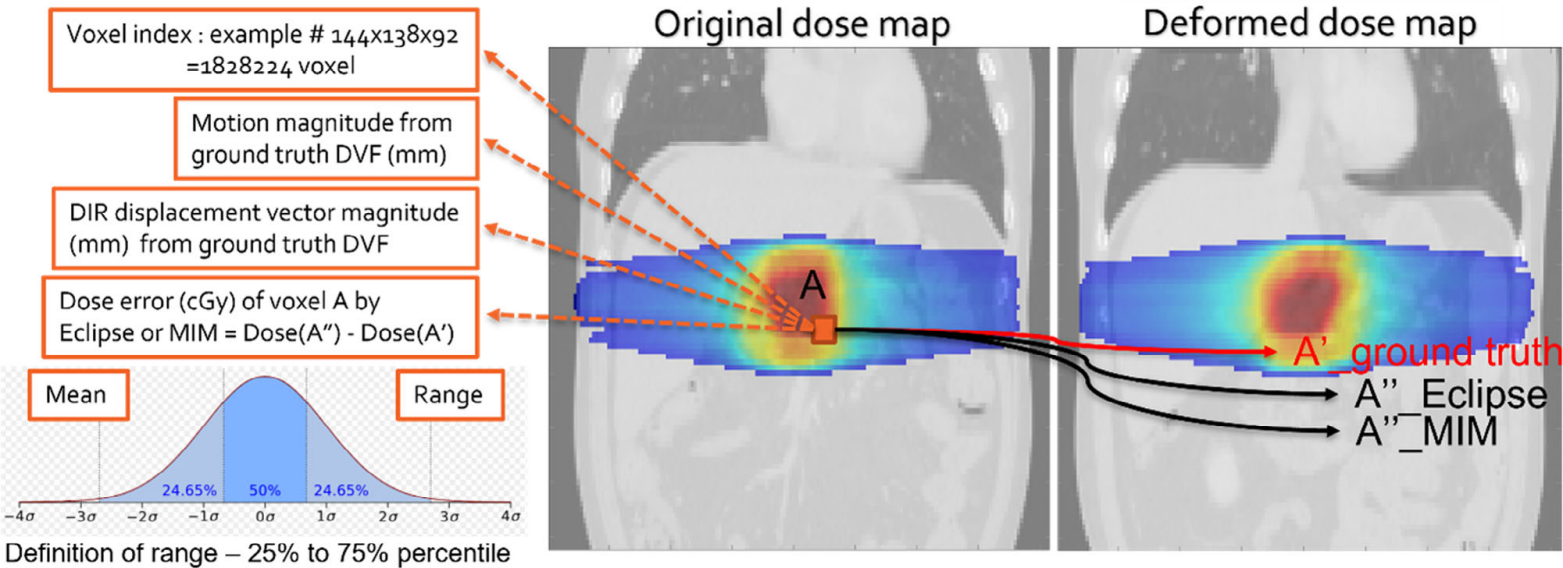
Geometric and dosimetric errors analysis of DVF for an example voxel.

To calculate the dose errors introduced by Eclipse DIR and MIM DIR, the following three steps were performed, as depicted in Figure 2: Step 1 involved finding the corresponding voxel (e.g., voxel A) on the original CT scan, dCT‐Eclipse (voxel A“_Eclipse), dCT‐MIM (voxel A”_MIM), and the ground‐truth deformed CT scan (voxel A'_Ground‐truth). In Step 2, the dose values were obtained for voxel A“_Eclipse from DM_Eclipse, for voxel A”_MIM from DM_MIM, and for voxel A'_Ground‐truth from DM_Ground‐truth. Finally, in Step 3, the dose error for Eclipse DIR was calculated by subtracting the dose at voxel A“_Eclipse in DM_Eclipse from the dose at voxel A'_Ground‐truth in DM_Ground‐truth, measured in centigray (cGy). Similarly, the dose error for MIM DIR was calculated by subtracting the dose at voxel A”_MIM in DM_MIM from the dose at voxel A'_Ground‐truth in DM_Ground‐truth, also measured in cGy. These steps were repeated for each voxel within the entire 3D original CT scan within the body contour to obtain the dose map errors using different DIR algorithms.

The distributions of dose readings for voxel A“_Eclipse from DM_Eclipse, voxel A”_MIM from DM_MIM, and voxel A'_Ground‐truth from DM_Ground‐truth were presented in histograms. Additionally, the distributions of dose errors at voxel A“_Eclipse, voxel A”_MIM, and voxel A'_Ground‐truth were also visualized in histograms.

The decision to recreate new deformed CT scans for both MIM and Eclipse was strategically chosen to enhance the accuracy of our DIR evaluations under simulated conditions that mimic clinical scenarios, including dose assessment with altered anatomy on treatment day during adaptive planning or dose accumulation during previous treatment evaluation.

As our analysis focused on RT, particular attention was given to critical organs and tumor. Therefore, our region of analysis was further restricted to include the GTV, small bowel, stomach, and duodenum. The same statistical analysis described previously was performed to obtain dose and dose error histograms within these regions.

In addition, several dose metrics were measured and tabulated for the GTV, including Dmax (maximum dose), Dmin (minimum dose), V100% (volume receiving 100% of the prescribed dose), and D95% (dose received by 95% of the GTV volume). Similarly, for the OARs, Dmax was measured.

**FIGURE 3:**
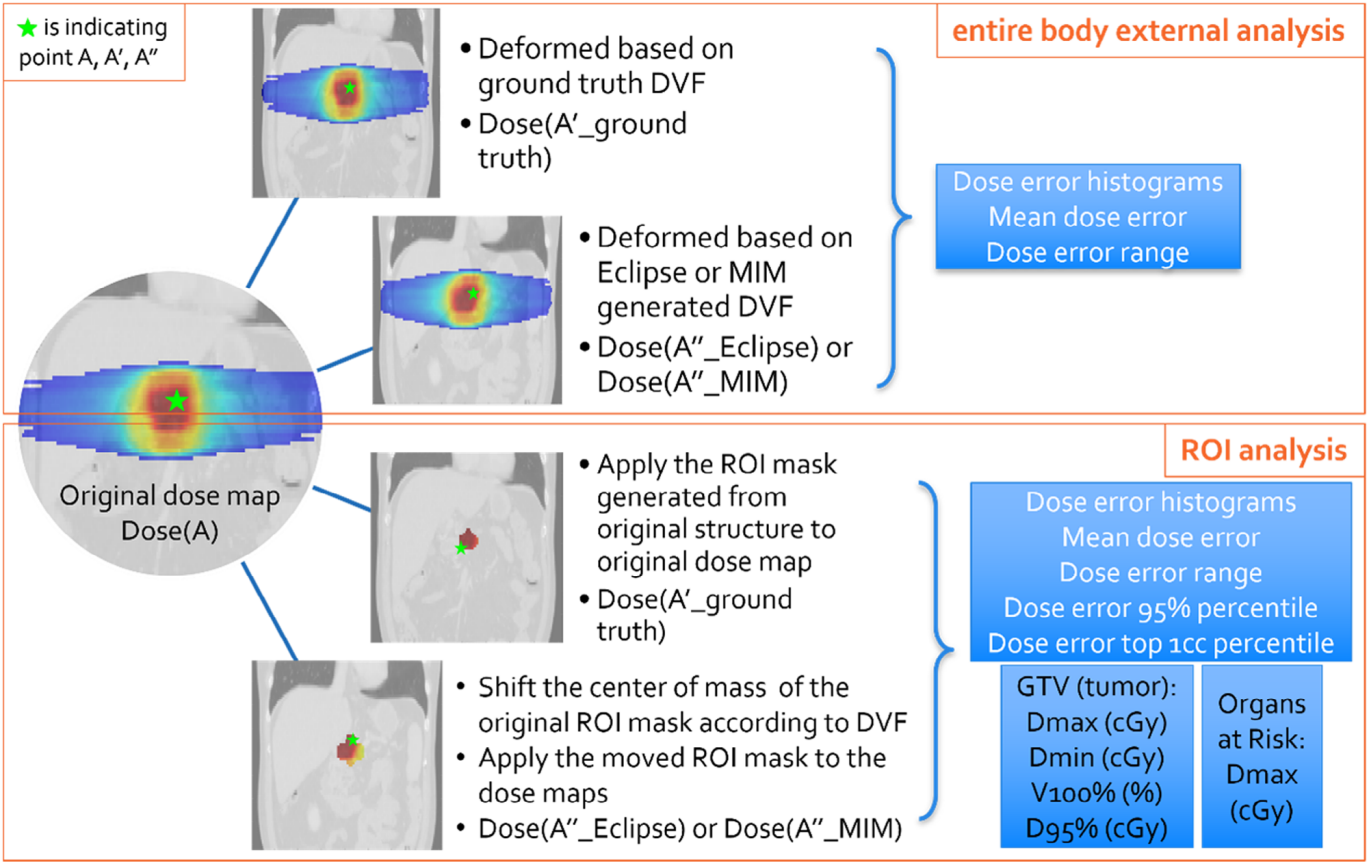
Analysis of DIR dosimetric impact in the entire body external and ROI. Dose map on original CT, ground‐truth deformed CT, Eclipse or MIM (demonstrating Eclipse as an example) deformed CT in the entire body external were demonstrated on the left, top first and second CT images, respectively. Dose map on ground‐truth deformed CT and Eclipse or MIM (demonstrating Eclipse as an example) deformed CT in ROI were demonstrated on bottom two CT images. Greens stars are marking the location of point A, A’ and A”.

Furthermore, six metrics were used for evaluation across all patients: ROI Dmax error, ROI Dmin error, ROI dose error mean, ROI dose error maximum, 95th percentile of ROI dose error, and ROI dose error for the top 1cc volume. The ROI here are referring to organs such as the stomach, duodenum, or small bowel, etc. These metrics were calculated as percentages relative to the prescription doses, which were listed in Table 1. If multiple prescription levels were used, the highest level was considered as the reference for the calculations. For each patient, these six metrics were calculated, and the average and standard deviation were determined. The process of analyzing the entire CT scan and the ROI is depicted in Figure 3.

For the aforementioned ROI analysis, first of all, the ROI motion was simulated based on both respiratory and digestive motion to create the “deformed CT.” For gastrointestinal (GI) ROIs that we focused on in our ROI analysis in the bottom image in Figure 3, the main organ motion was considered to be the digestive shifts (the 5 mm uniform shift) applied to the center of mass of the whole organs. Consequently, the ROI analysis was conducted on the shifted ROI to align with the simulated digestive motion.

Despite the digestive motion shift, all these organs, that is, ROI, remained within the planning CT. This analysis was conducted using voxels within the ROI on both the planning CT and the deformed/shifted CT. The dose distribution following deformation was recalculated on the deformed CT without modifying the dose distribution based on the DVF.

In the subsequent steps, when inputting the original planning CT and the respiratory deformed plus digestive motion‐shifted CT into DIR, the DIR algorithms detected both the deformation and the shifts we introduced.

### 2.3 Correlation between DIR magnitude error versus dose error

In addition to conducting patient‐specific QA, our study investigated the correlation between displacement magnitudes, DIR magnitude errors, and DIR introduced dose errors. As depicted in Figure 2, we extracted data from each voxel, including (1) the voxel index, (2) the ground truth displacement magnitude of the voxel, and (3) the DIR magnitude error measured for the voxel using Eclipse or MIM DIR. The voxel index served to distinguish each voxel from others, with the most anterior, left, and superior voxel on the 3D image assigned as the lowest voxel numbers. The ground truth displacement magnitude of each voxel was derived from the ground truth DVF and measured in millimeters (mm). The DIR magnitude error was calculated by subtracting the displacement magnitude value of the voxel from the exported DVFs of Eclipse or MIM with that from the ground truth DVF, also measured in mm.

Data extraction was performed for each voxel in the 3D CT scan, resulting in three sets of data, as described above, for each patient. To quantify the DIR magnitude errors, we binned the magnitudes for all voxels into bins based on the magnitude of each voxel's ground‐truth displacements, with a resolution of less than 1 mm. We employed this binning as a strategic approach to mitigate data noise and outliers. This method helps in managing variations within the data more effectively. While binning can introduce bias by grouping data, it was necessary in our study to ensure more stable and reliable statistical analysis, especially given the wide range of motion magnitudes observed. This approach allows us to reduce the impact of extreme values or anomalies on our overall analysis. Within each bin, the following parameters of DIR magnitude errors were obtained: (3a) the mean of the DIR magnitude errors for all voxels in the bin, and (3b) the range of the DIR magnitude errors for all voxels in the bin. The range of DIR magnitude errors was defined as the span (in mm) between the 25th percentile and the 75th percentile on the histogram of error distribution, as illustrated in the bottom right of Figure 2.

Using the aforementioned parameters, we investigated the relationship between the ground truth displacement magnitudes and the mean DIR magnitude errors using the ground truth magnitude of displacement (data groups^2^), and the mean ± range of DIR magnitude errors [data groups (3a and 3b)]. Additionally, we tested several other evaluators, such as the skew of the distribution of DIR magnitude errors. However, no clear trend or correlation (correlation coefficient < 0.6) between these evaluators and the ground truth magnitude of displacement (data group^2^) was observed.

Similar to the DIR magnitude error analysis, statistical analysis was performed to examine the correlation between displacement magnitudes and dose errors; all voxels within the original CT scan were sorted into bins based on motion magnitudes, which were obtained from the ground‐truth DVF. Same as before, for mitigating data noise and outliers, the spectrum of magnitude values for each voxel was sorted and divided into 50−100 magnitude bins. Each bin served as a container that encompassed hundreds of voxels with motion magnitudes falling within the range of that bin. The nominal magnitude of each bin represented the center value of its magnitude range.

Subsequently, the dose errors for each voxel within each magnitude bin were tracked. The mean dose error and dose error range (the span of 25th to 75th percentile on the dose error distribution histogram) were calculated for the voxels within each bin. This allowed for the characterization of the average dose error and the variability of dose errors within each magnitude bin.

The relationship between the mean dose errors (or dose error ranges) and the nominal magnitudes of each bin was investigated. By examining how the mean dose errors (or dose error ranges) varied with the nominal magnitudes, insights into the dependence of dose errors on motion magnitudes could be obtained.

The relationship between DIR magnitude error and dose error was also investigated. For each voxel in the ROI, we first calculated the DIR magnitude errors. Subsequently, all voxels were sorted and grouped into bins based on their DIR magnitude errors. Within each bin, the corresponding dose error for each voxel was calculated, creating a dose error pool for each bin. The mean and range of the dose errors in each bin were then determined. Thus, for each DIR magnitude error bin, we obtained the mean and range of its dose error.

## 3 RESULTS

### 3.1 Patient‐specific DIR performance analysis

#### 3.1.1 Geometric error analysis

**FIGURE 4:**
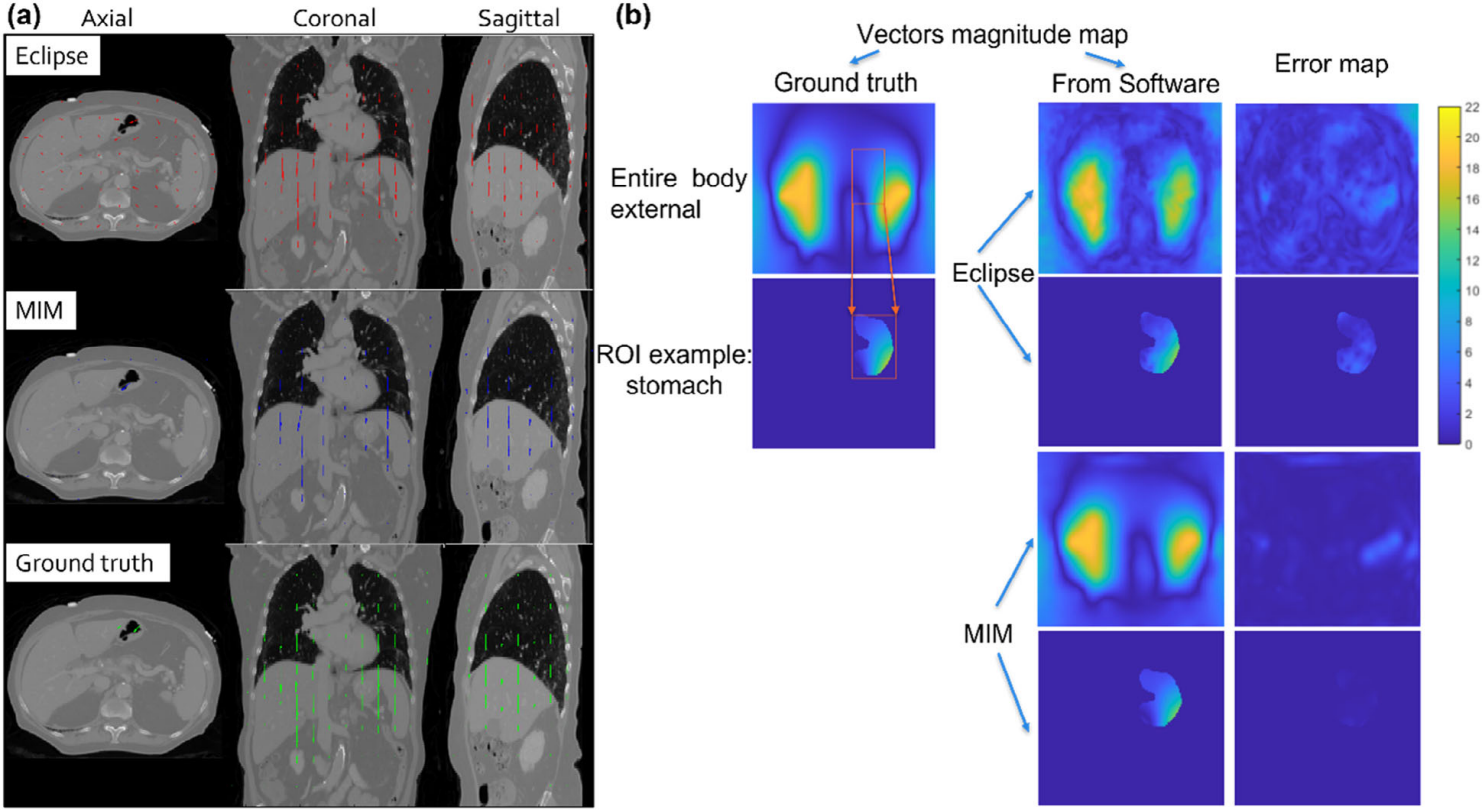
(a) Ground truth DVF, and DVF obtained with DIR in Eclipse and MIM; (b) Illustration of DVF magnitude maps on the entire body external and an example ROI of ground truth, Eclipse and MIM DIR, and the corresponding DIR magnitude error maps, in axial view.

The biomechanical model‐derived ground‐truth 3D DVF encompasses an FOV from the mid lung to the inferiormost boundary of the pancreas. The displacements within this region range from 0 to 38 mm. The ground‐truth DVF and the DVFs obtained using the DIR algorithms in Eclipse and MIM are presented in Figure 4a. These visualizations depict the axial, coronal, and sagittal views of the DVFs. Maps of the DIR DVFs and the corresponding DIR magnitude errors are displayed in Figure 4b. These maps are shown in axial view, covering both the entire CT scan and a specific ROI representing the stomach and duodenum. The DIR magnitude errors provide insight into the discrepancies between the DIR‐generated displacement vectors and the ground truth DVF within these areas.

The maximum and average measurements of ground‐truth displacement magnitudes for all voxels are presented in Table 2, including the measurements in the entire body external of the CT scan and the specified ROIs (stomach+duodenum and small bowel). It should be noted that larger discrepancies in displacement magnitudes are observed near the diaphragm compared to the mid lung or pancreas regions.

**TABLE 2:** Ground truth DVF motion range (maximum motion magnitudes) for the entire body external and ROIs, and mean value for the entire body external.

Ground truth DVF Max vector magnitude and mean (mm)	Pt 1	Pt 2	Pt 3	Pt 4	Pt 5
GTV (mean)	9.6 (4.1)	5.4 (2.1)	10.2 (5.6)	13.9 (4.0)	22.2 (7.4)
Stomach‐due (mean)	28.2 (13.5)	32.4 (8.3)	22.5 (10.2)	21.5 (7.0)	29.5 (12.6)
Small bowel (mean)	29.2 (12.6)	13.8 (3.2)	16.1 (9.0)	8.5 (5.0)	15.0 (9.2)
Entire body external (mean)	35.3 (7.0)	40.2 (4.1)	35.2 (5.4)	34.8 (6.4)	35.5 (4.8)

Abbreviation: Pt, patient.

**FIGURE 5:**
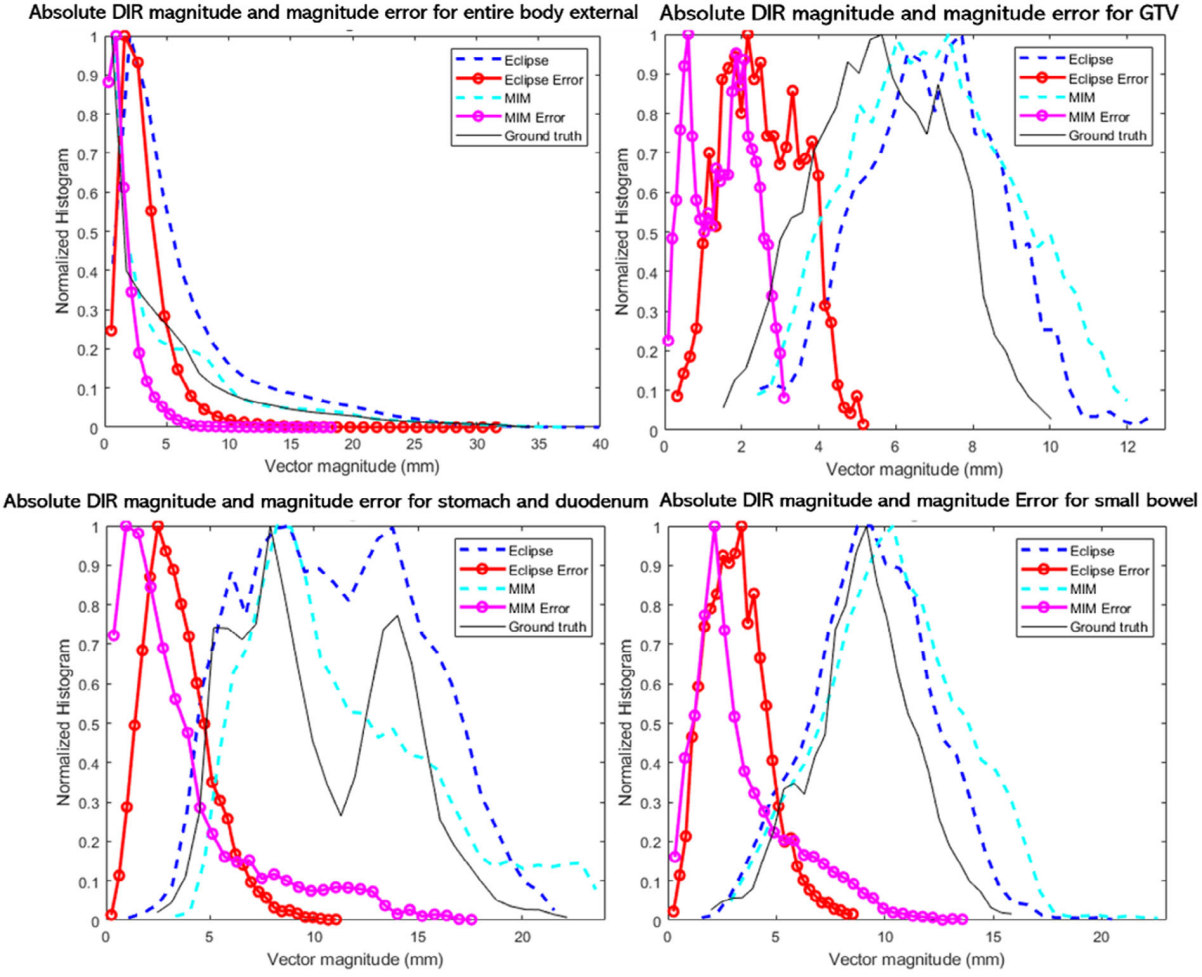
Histograms of DVF vector magnitude distribution (in dark blue for Eclipse, light blue for MIM, and black for ground truth) and the DIR magnitude error distribution (in red for Eclipse and purple for MIM). Same histograms are shown for entire body external analysis, GTV, stomach+duodenum, and small bowel analysis.

Figure 5 displays the histograms of ground‐truth displacement magnitudes, as well as the magnitude of displacement vectors obtained from the Eclipse and MIM DIR algorithms and the corresponding DIR error histograms.

Table 3 reports the mean values of the DIR magnitude errors with Eclipse or MIM, for individual patients and on average over all five patients. Similarly, the table also provides the 90th percentile measurements of the DIR magnitude error distribution. Across the entire CT scan, Eclipse DIR exhibits slightly larger magnitude errors compared to MIM DIR. Among the ROIs, patient #1 demonstrates a different error pattern compared to the other patients, with significantly larger DIR magnitude errors observed with Eclipse DIR compared to MIM DIR. This patient could be considered a challenging case within the patient group, potentially indicating an unreliable performance of the DIR algorithms in Eclipse and MIM. No significant inter‐observer differences were observed.

**TABLE 3:** Mean value of the DIR magnitude error and 90% percentile of the error distribution on the entire body external and at ROIs.

Patient #		1		2		3		4		5		Average ± SD	
Platforms		Eclipse	MIM	Eclipse	MIM	Eclipse	MIM	Eclipse	MIM	Eclipse	MIM	Eclipse	MIM
DIR magnitude error mean (mm)	GTV	4.5	0.9	2.0	0.4	2.5	1.5	2.7	0.6	2.3	2.2	2.8 ± 1.0	1.1 ± 0.7
	Stomach‐due	12.4	2.2	4.1	2.8	3.4	3.6	3.1	0.8	3.1	2.9	5.2 ± 4.0	2.5 ± 1.0
	Small bowel	12.1	1.8	2.3	1.8	3.2	3.4	2.9	2.0	3.5	4.3	4.8 ± 4.1	2.7 ± 1.1
	entire body external	11.6	1.4	3.1	0.8	3.1	1.5	3.9	1.0	2.9	2.0	4.9 ± 3.8	1.3 ± 0.5
90% percentile (mm)	GTV	6.7	1.6	3.0	0.8	3.9	2.5	4.3	0.8	3.9	5.0	4.4 ± 1.4	2.1 ± 1.7
	Stomach‐due	29.6	4.9	8.5	7.1	5.6	8.7	5.0	2.0	5.1	6.3	10.8 ± 10.6	5.8 ± 2.5
	Small bowel	28.4	4.0	4.3	4.7	5.0	7.0	4.7	4.7	5.5	8.9	9.6 ± 10.5	5.9 ± 2.0
	entire body external	22.5	2.8	6.1	1.7	5.4	3.1	6.8	2.1	5.9	4.2	9.3 ± 7.4	2.8 ± 1.0

Abbreviation: SD, standard deviation.

The relationships between the ground truth displacement magnitudes and mean DIR magnitude errors, as well as the range of the DIR magnitude errors over the entire body external, was depicted on the top part of Table 4, as in correlation coefficient. The correlation coefficients between the mean or range of the DIR magnitude errors and the ground truth displacement magnitudes are also listed. Five curves in different colors show the data from five different patients in our study. The results indicate that as the ground truth displacement magnitudes increase, both the mean and range of the DIR magnitude errors tend to increase. The range of the DIR magnitude error exhibits a positive correlation with the ground truth displacement magnitudes, with an average correlation coefficient of 0.8 for Eclipse and 0.9 for MIM across all patients. The mean DIR magnitude error also shows a positive correlation with the ground truth displacement magnitudes, and the mean correlation is 0.8 for Eclipse, and 0.9 for MIM.

**TABLE 4:** Summary of the correlation between the DIR magnitude errors versus the ground truth displacement vector magnitudes (top two rows with numbers, first row for Eclispe and second row for MIM); the correlation between the DIR‐introduced dose errors versus the ground truth displacement vector magnitudes (middle two rows with numbers); and the correlation between the DIR‐introduced dose errors versus the displacement vector magnitude errors (bottom two rows with numbers).

DIR vector magnitude error (mean or range) vs. DIR vector magnitude								
Correlation coefficients	Entire CT		GTV		Small bowel		Stomach+duodenum	
	error mean	error range	error mean	error range	error mean	error range	error mean	error range
Eclipse	0.8	0.8	0.1	0.4	0.4	0.2	0.7	0.5
MIM	0.9	0.9	0.8	0.3	0.4	0.4	0.4	0.2

Dose error (mean or range) vs. DIR vector magnitude								
Correlation coefficients	Entire CT		GTV		Small bowel		Stomach+duodenum	
	error mean	error range	error mean	error range	error mean	error range	error mean	error range
Eclipse	0.6	0.8	0.0	0.1	0.1	0.1	0.3	0.3
MIM	0.2	0.8	−0.1	0.4	−0.1	0.2	0.0	0.2

Dose error (mean or range) vs. DIR vector magnitude error								
Correlation coefficients	Entire CT		GTV		Small bowel		Stomach+duodenum	
	error mean	error range	error mean	error range	error mean	error range	error mean	error range
Eclipse	0.4	0.8	−0.1	0.8	0.6	0.9	0.2	0.9
MIM	−0.8	0.9	−0.8	0.9	1.0	0.9	−0.6	0.9

The correlation data for the entire CT, GTV, small bowel, and stomach+duodenum are listed.

#### 3.1.2 Dosimetric error analysis

**FIGURE 6:**
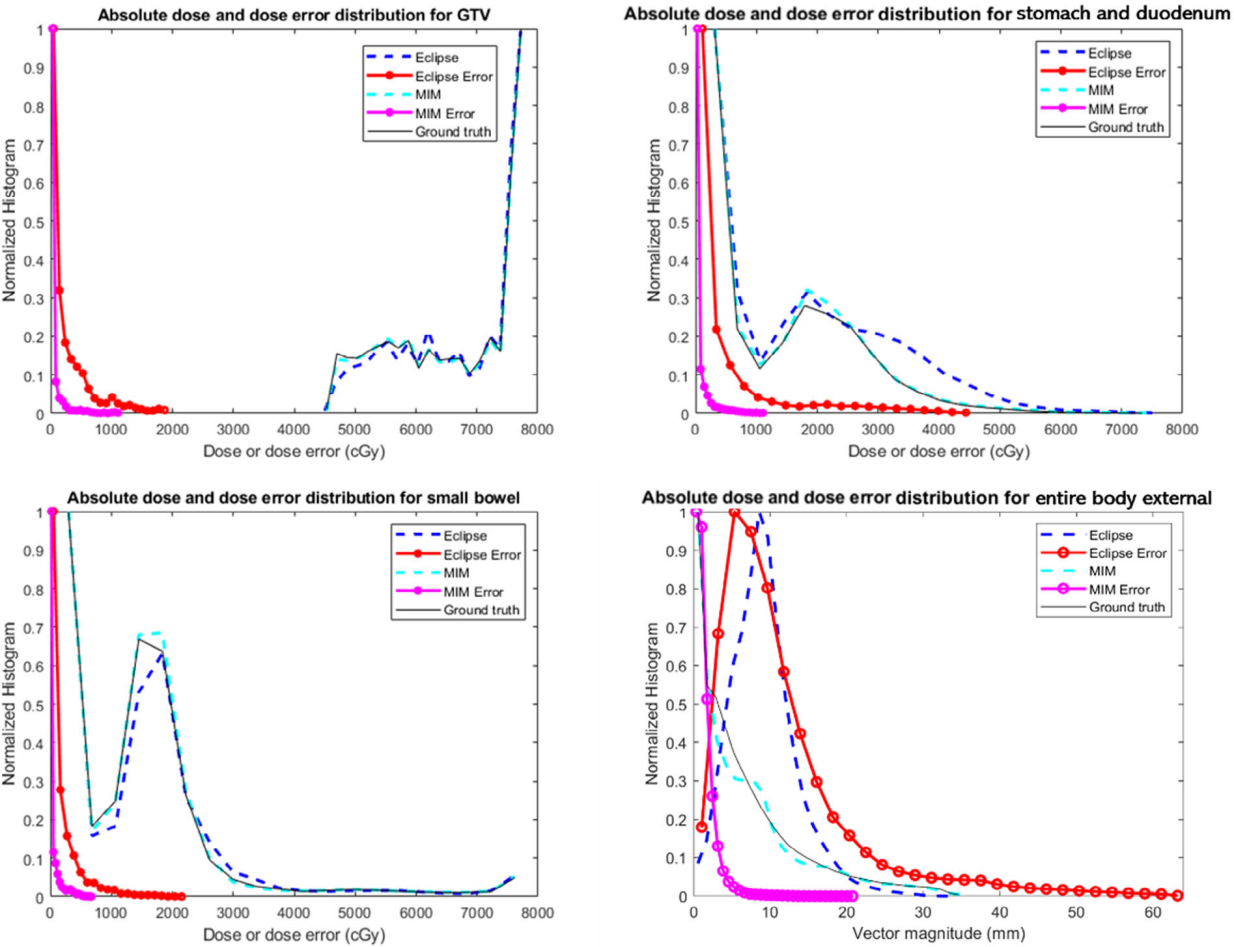
Histograms of dose distribution (in dark blue for Eclipse, light blue for MIM, and black for ground truth) and dose error distribution introduced by DIR (in red for Eclipse and purple for MIM). Same histograms are shown for entire body external analysis, GTV, stomach+duodenum, and small bowel analysis.

The dose distribution histograms on ground‐truth deformed CT, Eclipse‐deformed CT, and MIM‐deformed CT are illustrated in black, dark blue, and light blue curves in Figure 6. The DIR‐introduced dose error distribution in histograms is shown in the same figure in red and purple for Eclipse and MIM, respectively.

**FIGURE 7:**
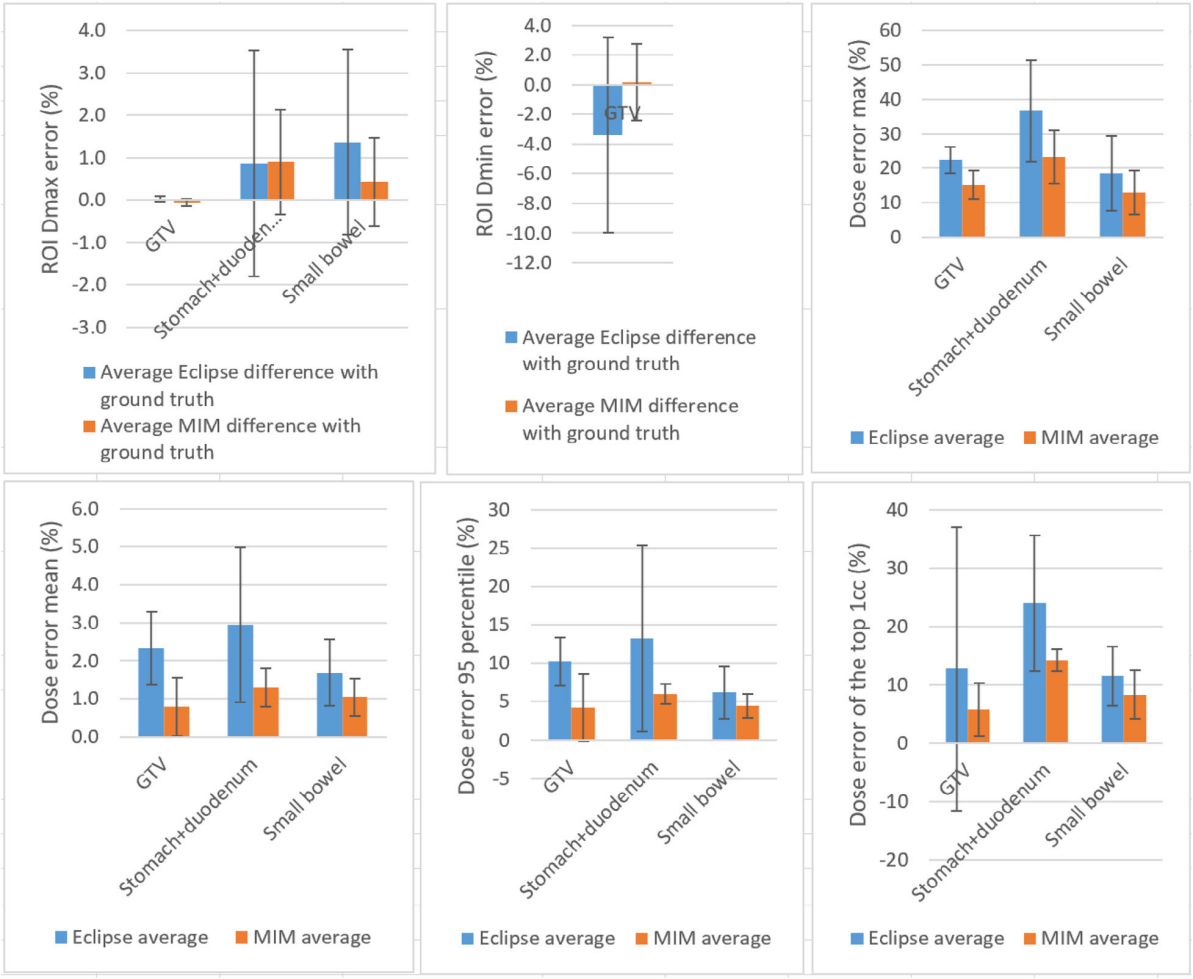
Mean (blue and orange bar) and standard deviation (error bar) over all patients of the DIR introduced ROI dose error in terms of—error of Dmax, error of Dmin, maximum of dose error, mean of dose error, 95 percentile of dose error, and dose error of the top 1cc of ROI. All values shown in percentage in reference to highest prescription of each patient.

The measurements of Dmax, Dmin, V100%, and D95% for GTV, as well as Dmax for stomach+duodenum, small bowel, were obtained with reference to the highest prescription or the ROI total volume of each patient. The error was calculated comparing the measurements made on DM_Eclipse and DM_Ground‐truth, or DM_MIM and DM_Ground‐truth. The mean error across all patients was minimal. Dmax, Dmin, V100%, and D95% dose error for GTV are all within 4% using Eclipse DIR, and within 0.2% using MIM DIR. Dmax dose error for OARs are all within 2% using Eclipse DIR, and within 1% using MIM DIR. Figure 7 presents the average measurements, along with error bars representing the standard deviation, for ROI Dmax error, ROI Dmin error, ROI dose error mean, ROI dose error maximum, 95th percentile of ROI dose error, and ROI dose error of the top 1cc, over all patients. ROI Dmax error is the mean Dmax error for each ROI, over all patients. Dose error max (per ROI) is the maximum of the ROI voxel‐to‐voxel dose errors. Errors are calculated with sign and without calculating the absolute error values.

### 3.2 Correlation between DIR magnitude error versus dose error

The relationships between the ground truth displacement magnitudes and the mean DIR magnitude errors, as well as the range of the DIR magnitude errors, over the stomach+duodenum, small bowel, and GTV are presented in the top part of Table 4.

The relationships between the ground truth displacement magnitudes and the dose error mean, or dose error range for the stomach+duodenum, small bowel, and GTV are displayed in the middle part of Table 4. Additionally, the relationships between the DIR magnitude errors and the dose error mean, or dose error range for the stomach+duodenum, small bowel, and GTV are shown in the bottom part of Table 4.

Based on the analysis presented in the top and middle part of Table 4, it can be observed that there is a weak correlation between the DIR magnitudes and both the DIR magnitude error and dose error. However, in the bottom part of Table 4, a stronger correlation is observed between the DIR magnitude errors and the DIR‐introduced dose error ranges, with correlation coefficients greater than 0.8. This indicates that higher DIR vector magnitude errors are associated with larger DIR‐introduced dose error ranges. Data for each patient were not listed separately in Table 4, as the trends observed were similar across all patients.

## 4 DISCUSSION

Following AAPM TG‐132 guidelines, this study has developed a patient‐specific QA process to assess the accuracy of DIR for abdomen sites. The methodology established in this study can be applied to evaluate the DIR accuracy for individual patients. This study is vital, as it explores the direct impact of DIR accuracy on dose calculation in RT, especially for challenging abdominal images. By developing a novel QA tool and focusing on the recalculation of dose on deformed images, this research aims to improve the precision of RT, ultimately enhancing patient safety and treatment outcomes. More specifically, the reason why this study is important for RT lays in the following five‐folds:

1. Importance of DIR accuracy: In RT, the precision of dose delivery is paramount. DIR is used to align images taken at different times or under varying conditions, allowing for accurate tracking of anatomical changes over time. This is particularly crucial in abdominal imaging, where structures are prone to movement and deformation due to respiratory and digestive motions. The accuracy of DIR directly impacts the calculation of the radiation dose accumulated over multiple sessions. A minor error in DIR can lead to significant discrepancies in dose calculations, potentially affecting treatment outcomes.
2. Challenges in assessing DIR performance: One of the primary challenges in evaluating DIR's effectiveness is the lack of a reliable ground truth, especially for abdominal images. These images often suffer from poor soft‐tissue contrast and are susceptible to severe motion artifacts, making it difficult to ascertain the true displacement of tissues. Without a standard for comparison, assessing the geometric accuracy of DIR is challenging.
3. Impact on dose calculation: Despite the importance of DIR in RT, the extent to which its accuracy affects dose calculation has not been thoroughly investigated. This uncertainty can lead to errors in treatment planning and delivery, which could compromise patient safety and treatment efficacy. This recalculation of dose on deformed images process is crucial, as it directly links DIR accuracy to potential dose miscalculations, providing a clear picture of how DIR inaccuracies can impact patient treatment.
4. Development of a novel QA tool: To address these challenges, this study introduces a novel patient‐specific QA tool. This tool leverages a digital phantom recommended by AAPM Task Group 132 for DIR evaluation. By using patient‐specific planning CT images and inducing deformations through a biomechanical motion model, the tool can simulate realistic anatomical changes.
5. Preliminary investigation and patient‐specific QA: The procedure outlined in this study has been preliminarily applied to data from five pancreatic cancer patients. This approach serves as a critical tool for evaluating patient‐specific dose uncertainties introduced by DIR, underscoring the importance of accurate DIR for effective RT planning and delivery.

This study offers several key advantages. First, it provides an evaluation method for major DIR platforms commonly used in clinical practice. Second, the evaluations were performed for anatomical sites with significant deformations, including lung and abdomen, considering both respiratory and digestive motions. Third, pancreatic cancer patients’ data were evaluated. Lastly, reliable ground‐truth was employed for DIR assessment in this study.

It is noteworthy to highlight our study design for evaluating the dose error introduced by DIR. While typically the dose could be calculated directly on ReCT or CBCT images and then mapped back to the planning CT using DIR, our approach of generating new deformed CT scans helps to isolate the influence of DIR errors on dose calculations without the additional variabilities introduced by different imaging modalities. DIR applied to register CBCT and CT may result in a different error pattern and magnitude. Based on the clinical scenarios, we focus on—previous treatment evaluation and adaptive planning. The image registration will be in between the same modality—previous treatment course planning CT versus current course planning CT for previous treatment evaluation; and planning CT from re‐simulation versus planning CT from existing simulation.

To validate the introduced digestive motion in our study, we conducted a comparison with a surface analysis presented in the study by Han et al.^17^ The study by Han et al. involved a dataset of 40 pancreatic cancer patients, and we focused on a subset of five patients from this dataset for our investigation. Han et al. defined their ground‐truth from physician‐drawn ROI (stomach+duodenum and small bowel) contours on both the planning CT and daily CBCT scans. For each point A on the planning CT contour, the corresponding point A' on the CBCT contour with the shortest distance was identified. The vector from point A to point A' was defined as the ground‐truth DVF for point A. Subsequently, Han et al. performed MIM DIR to register the planning CT onto the CBCT, and the resulting DVF was compared with the ground‐truth DVF. In their study, Han et al. utilized a deep learning‐based DIR technique to generate the DVF, which represents a more and precise approach to capturing and analyzing anatomical changes over time. The ground truth DVF from Han is from surface vectors with the shortest distance. But their DIR is utilized a deep learning‐based DIR technique to derive the DIR DVF, which is evaluated in their study. The deep learning based DIR in their study was evaluated and compared with the surface‐vector ground truth DVF.

The resulting mean MIM DIR displacement magnitude errors on the ROI contour surfaces were compared with those calculated using our method on the same patient cohort. The comparison results are tabulated in Table 5. Notably, our DIR calculation method was applied to the entire ROI volume for the results tabulated in Table 5 (pt1‐pt5). In contrast, Han et al. performed their measurements only on the ROI surfaces for the results listed in Table 5 (pt1‐pt5). The data presented in Table 5 indicate that the DIR errors measured within the abdominal ROI in our study are consistent with those reported in the study by Han et al.

**TABLE 5:** Comparison of the mean surface DIR displacement errors from Han's study and mean volumetric DIR displacement errors measured in our study.

Patient	Mean MIM DIR displacement magnitude errors on the ROI contour surfaces from (a) our study vs. (b) from Han, MedPhys 2021 (mm)	Stomach‐due	Small bowel
Pt1	(a)	2.0	1.8
	(b)	5.2	3.9
	(a)‐(b)	−3.2	−2.1
Pt2	(a)	2.7	1.7
	(b)	4.8	3.7
	(a)‐(b)	−2.1	−2.0
Pt3	(a)	3.1	3.3
	(b)	4.2	3.0
	(a)‐(b)	−1.1	0.3
Pt4	(a)	1.2	1.9
	(b)	4.2	4.7
	(a)‐(b)	−3.0	−2.8
Pt5	(a)	2.6	2.9
	(b)	5.2	4.1
	(a)‐(b)	−2.6	−1.2
Average difference	(a)‐(b)	−2.4 ± 0.8	−1.6 ± 1.2

Category (a) represents the volumetric error mean in mm from our study vs. category; (b) represents the surface error mean in mm from Han, MedPhys 2021.^17^

The patient‐specific QA process was well‐established in our study. However, there are areas in which this study can be further improved. First, the evaluation and analysis can be expanded to examine the accuracy of other DIR algorithms, beyond Eclipse and MIM. Second, the number of patients included in this pilot study is limited. Further investigation and validation with a larger patient are desirable in order to enhance the reliability and generalizability of the findings, especially for the correlation analysis. It is noteworthy that certain curves in the figure exhibit significantly larger errors compared to the other patients. This discrepancy may be attributed to greater motion displacement observed in the stomach+duodenum and small bowel of this particular patient relative to the other patients. In this study, all five patients had their GTV located near the spine, where less motion was simulated. As a result, the correlation coefficients in Table 4 display similar trends across all patients. To establish a more distinct pattern and achieve a comprehensive evaluation, it would be beneficial to include a larger dataset encompassing data from additional patients. In future studies, we plan to incorporate these considerations to further enhance our patient‐specific QA for DIR evaluation.

## 5 CONCLUSION

A preliminary evaluation was conducted to assess the accuracy of DIR algorithms using a realistic biomechanical model generated ground‐truth. Geometric error and dosimetric impact of the DIR algorithms were evaluated at abdomen for pancreatic cancer patients. A patient‐specific DIR QA workflow was developed, showing potential for future implementation as a clinical workflow.

## AUTHOR CONTRIBUTIONS

Yilin Liu, Pengpeng Zhang, and Sadegh Alam conceived the original idea. Laura I. Cerviño Arriba and Pengpeng Zhang supervised the project. LiCheng Kuo, Yu‐chi Hu, Wei Lu helped supervised the project. Yilin Liu and Jun Hong carried out the experiment. Yilin Liu wrote the manuscript with support from Pengpeng Zhang, Jun Hong, and Wei Lu.

## CONFLICT OF INTEREST STATEMENT

The authors declare no conflicts of interest.
